# Death by dengue fever in a Brazilian child: a case report

**DOI:** 10.1186/1756-0500-7-855

**Published:** 2014-11-27

**Authors:** Rafael Henrique Machado Sacramento, Deborah Nunes de Melo Braga, Franciane Fardin Sacramento, Fernanda Montenegro de Carvalho Araújo, Antônio Afonso Bezerra Lima, Margarida Maria de Lima Pompeu, Danielle Malta Lima, Luciano Pamplona de Góes Cavalcanti

**Affiliations:** Equipe Indígena de Saúde do Ministério da Saúde - EMSI/DSEI-CE/SESAI/MS, Fortaleza, Brazil; Programa de Pós-graduação em Patologia da Universidade Federal do Ceará, Fortaleza, Brazil; Laboratório Central de Saúde Pública do Ceará, Fortaleza, Brazil; Hospital São José de Doenças Infecciosas, Fortaleza, Brazil; Departamento de Patologia da Universidade Federal do Ceará, Fortaleza, Brazil; Universidade de Fortaleza, Fortaleza, Brazil; Department of Community Health, School of Medicine, Federal University of Ceará, St. Prof. Costa Mendes 1608, 5th Floor, Fortaleza, CE 60430-140 Brazil

**Keywords:** Autopsy, Death, Dengue, Indigenous

## Abstract

**Background:**

Dengue is an important worldwide public health problem, and continues to spread in Brazil. This article presents the first Brazilian case report of the death of an indigenous child by dengue fever.

**Case presentation:**

In August 2013, a child aged 2 years and from the Tremembé ethnic group, who was previously healthy with no complaints, suddenly presented intense crying, precordial pain, and general malaise. A few minutes after these non-specific symptoms, the patient started tonic–clonic convulsions and had cyanosis, a substantial increase in body temperature to the touch, cold sudoresis, sphincter relaxation, and unconsciousness. This situation remained for 15 minutes, progressing to respiratory insufficiency, with consequent absence of peripheral pulses. Death was confirmed approximately 40 minutes after the first symptoms. An autopsy was performed using the usual techniques. Immunohistochemistry was positive for dengue, and microscopic examination indicated micro perivascular edema and cerebral hemorrhage.

**Conclusion:**

Considering that the death occurred during the major endemic seasonal period for dengue fever, primary clinical evidence suggestive of viral infection presenting with sudden and quick death, and positive immunohistochemistry results, the case was closed as severe dengue fever. Clinicians must consider dengue as a diagnostic hypothesis among the indigenous population in Brazil.

## Background

Dengue is an important worldwide public health problem, and is the most common arthropod-borne viral illness in humans [1]. Several populations have been affected, with higher mortality among children, the elderly, and patients with comorbidities [1, 2]. Among these vulnerable populations, indigenous people are typically excluded from Brazilian research [3]. Brazil has one of the largest indigenous populations in the world, encompassing approximately 800,000 people, including 230 ethnic groups and 180 different languages [4–6]. However, there are no scientific articles or reports about dengue fever in these populations. This article presents the first Brazilian case report of the death of an indigenous child by dengue fever.

## Case presentation

The patient was a child aged 2 years and a member of the Tremembé ethnic group indigenous to northeastern Brazil. In August 2013, the patient, who was living in the State of Ceará and was previously healthy with no complaints, suddenly presented intense crying, precordial pain and general malaise. A few minutes after these non-specific symptoms, the patient started tonic–clonic convulsions and had cyanosis, a substantial increase in body temperature to the touch, cold sudoresis, sphincter relaxation, and unconsciousness. This situation remained for 15 minutes, progressing to respiratory insufficiency, with consequent absence of peripheral pulses as the patient was on the way to the hospital.

At the emergency room in a local hospital, after resuscitation maneuvers without success and without any biotic response, death was confirmed, approximately 40 minutes after the first symptoms. Because the child was under medical supervision by the Indigenous Health team and was apparently healthy, without any evidence of violence, poisoning or previous diseases, the family was consulted about the necessity to submit the body to autopsy. After documented authorization, the Death Verification Service (DVS) received the case.

During the anamnese, the family said to the DVS pathologist that the child had presented cold-like symptoms.

Autopsy was performed under usual techniques. The pericardium was cut and the aorta punctured for blood sample collection. A total of 10 mL of blood was collected with a sterilized syringe and needles, without the use of anticoagulant. The skullcap was removed, the dura mater was removed and 2 mL of cerebrospinal fluid (CSF) was collected from the subdural space. For this procedure, all materials were sterilized. Samples of brain, heart, lungs, liver, and spleen (1–2 cm wide) were collected *in situ* with sterilized equipment (tweezers and scalpel). The fragments were preserved in two sterile plastic bottles, with and without formaldehyde.

Considering that the death occurred during the major seasonal period for dengue fever, samples without formaldehyde were sent for molecular analysis, and samples with formaldehyde were sent for histological techniques and immunohistochemistry for dengue diagnosis. All samples without formaldehyde were sent to Central Public Health Laboratory of Ceará (LACEN-CE) to perform diagnostic tests for anti-dengue IgM antibody, non-structural 1 (NS1) antigen, viral isolation, and real time PCR (RT-PCR). All tests were negative for IgM antibody, NS1 antigen, viral isolation and RT-PCR. The sample for immunohistochemistry was sent to the national reference laboratory at the Evandro Chagas Institute for further analysis.

The autopsy showed a male child, eutrophic, well nourished, presenting cyanosis on the lips and nails, with nasal white secretion compatible with food debris. The brain showed a slight flattening of spins and herniation of the cerebellar tonsils (weight: 1.3 kg), and the lungs were aerated with small purplish areas. The heart was slightly smooth and pale. The liver, spleen, kidneys, and adrenal glands contained congested aspects.

**Figure 1:**
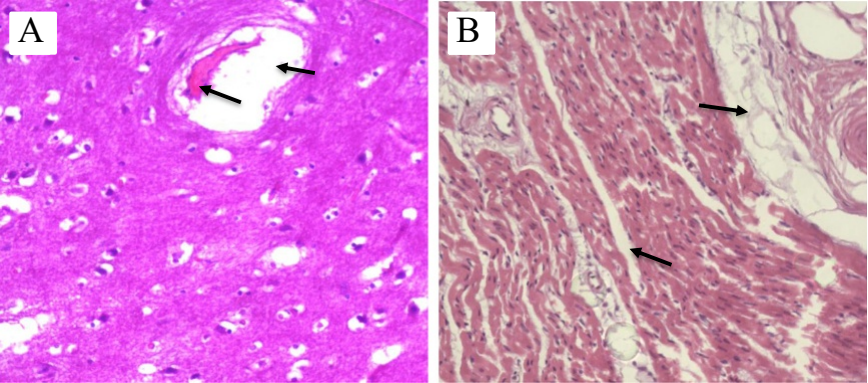
**Photomicrographs showing evidence of plasma linkage. A)** Section of the brain (brainstem) showing marked edema around the vascular structure; **B)** Section of myocardium showing perivascular and interstitial edema, dissociating bundles of muscle fibers.

**Figure 2:**
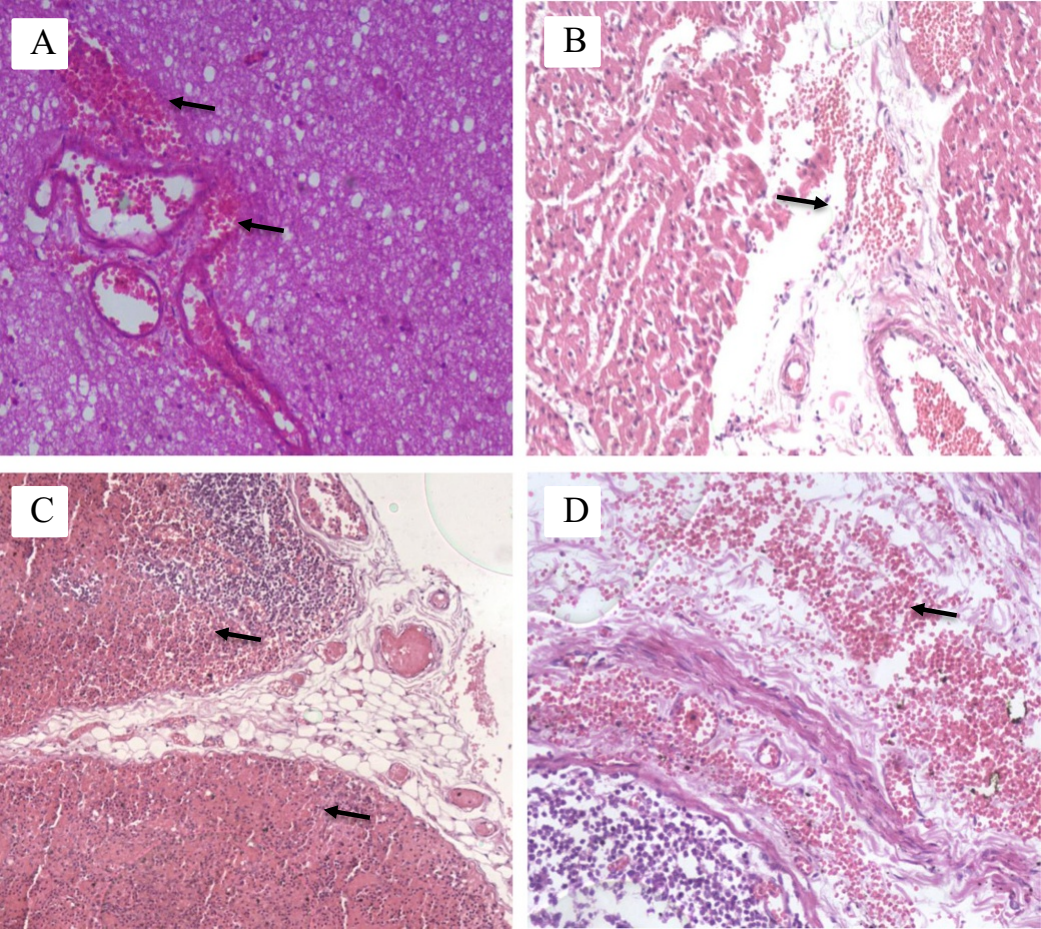
**Photomicrographs showing microscopic foci of hemorrhage. A)** Microhemorrhages around vascular structures of the brain; **B)** Perivascular hemorrhage in the myocardium; **C)** Hemorrhage in mediastinal lymph node; **D)** Hemorrhage in the capsule of the thymus.

**Figure 3:**
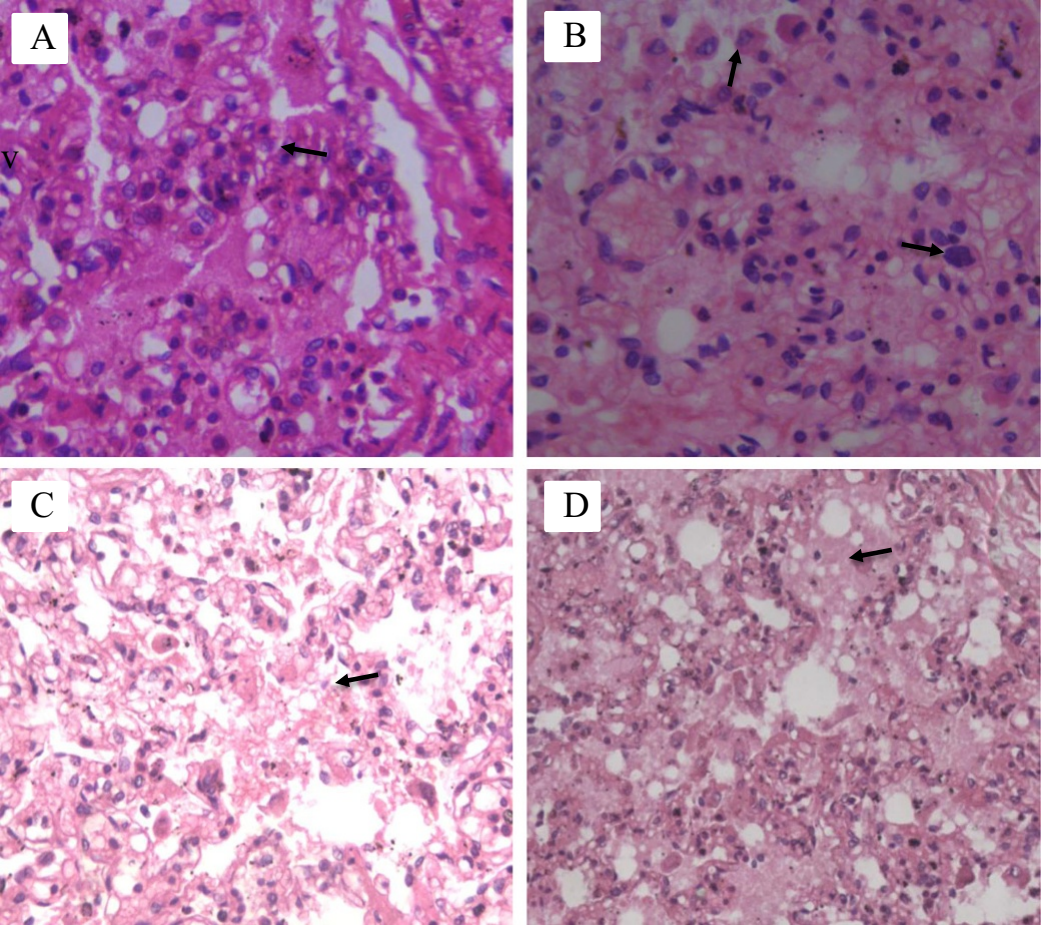
**Photomicrographs showing lung. A)** Thickening of the alveolar septa due to congestion and infiltration of lymphocytes scattered; **B)** Septal atypical mononuclear cells and pneumocytes in the alveolar lumen; **C)** and **D)** Edema and intra-alveolar hemorrhage.

Under microscopic examination, perivascular edema and interstitial edema were revealed in all organs; however, they were more pronounced in the meninges, brain, and the muscle fibers of the myocardium (Figure 1A, B). Focal microhemorrhages were shown in the perivascular space, mainly in the cerebral stem and basal ganglia, lymph nodes of mediastinum, thymus capsule, and myocardium (Figure 2A, B, C, D). The alveolar septum of both lungs was slightly thickened by capillary congestion and lymphocyte infiltration. Flaking pneumocytes mixed with erythrocytes and edema fluids were identified inside the alveolar spaces; however, these alterations were focal. Mononuclear cells with large and irregular nuclei were visualized in alveolar septa (Figure 3A, B, C, D). Accumulation of intracellular fluid, represented by hydropic degeneration of hepatocytes, was seen diffusely in the liver sections. Focal segmental tubular necrosis and scanty eosinophilic material within Bowman’s space was found in both kidneys. Immunohistochemistry showed a positive result for dengue, with micro perivascular edema and cerebral hemorrhage.

In the face of the favorable epidemiological condition with dengue epidemics in nearby regions, primary clinical evidence suggestive of viral infection presenting with sudden and quick death, and positive immunohistochemistry results, the case was found to be dengue fever and classified as severe dengue.

## Conclusions

We present the first report of an indigenous child death caused by dengue fever in Brazil. It is possible that other deaths have occurred before this, but the challenges of research with indigenous populations leads to under-notification and neglect. An initial dengue fever case in children could be indistinguishable from a regular cold [7, 8]. The hemorrhage and cerebral edema seen in this case may be caused by brain hypoperfusion and consequent central anoxia, leading to brain infarction. The cardiac edema with dissociation of muscular fibers, within plasmatic fluid leak as usual with severe presentations of dengue fever, may disturb conductions, promoting sudden death. This condition, in several reports, is a consequence of cardiac events.

Evidence of a direct lesion of the myocardium by dengue virus and presence of the virus in CSF have been described [9], but there was no significant inflammatory infiltration or necrosis in these organs to suggest direct lesions by dengue virus in this case, and there was also no evidence of the presence of dengue virus in CSF.

The short time evolution and fast lethal conclusion stands out in this case as a severe manifestation of dengue. Strong evidence links co-infections and pre-morbidity as major risk factors for death [10–13], but in this case no special conditions were present, besides the patient being a child. Childhood has been related to several atypical and severe manifestations with dengue, including sudden death [9, 14].

Immunohistochemistry was the only positive test, and all of the molecular techniques were negative. This situation could be because the samples were collected more than 12 hours after the death and the viral RNA could have degraded. The clinical manifestations took less than 24 hours, which might explain the absence of detectable IgM antibodies.

It is estimated that at the arrival of European settlers in Brazil there were more than 5 million indigenous individuals, which has reduced to approximately 800,000 in 2010, according to the last census of the Brazilian Institute of Geography and Statistics [6]. Thus, every effort to identify diseases and preventable disorders should be committed to avoid, or at least reduce, deaths in these historically disadvantaged populations, especially with regard to infant deaths.

Brazilian indigenous health services have gone through several changes since the original Service of Indigenous Protection (SPI). The SPI was ended in 1943, and indigenous health services then became part of the National Foundation of Indigenous (FUNAI), part of the National Health Foundation. The recently-established Special Secretary of Indigenous Health, a subsystem of FUNAI, manages the health services that should be the right of these populations.

The Tremembé indigenous group remained in silence for over two centuries, suffering persecution from state and society. They started to reveal themselves in 1980, living a constant process of re-ethnization and re-discovery of their origins, valuing the remnants of the facts that characterize them as indigenous [15]. Despite losing many of the phenotypes that would identify them with the Amazonian indigenous, they keep rites, customs, and common habits to indigenous people of northeastern Brazil, and reproduce their indigenous self-identification across generations.

We recognize the bureaucracy involved in working with indigenous health in Brazil, but we believe that this understanding must be encouraged to provide better and appropriate health services to their needs. Recognizing that we have dengue among indigenous populations reminds us of the need to train health professionals who work in indigenous health and make them aware of this differential diagnosis. This makes it necessary to understand that other dengue cases may be occurring in diverse areas in Brazil where there are indigenous populations, and require interventions and specific public policy to address this possible burden of disease.

## Consent

Written informed consent was obtained from the patient’s parents for publication of this Case Report and any accompanying images. A copy of the written consent is available for review by the Editor-in-Chief of this journal.
